# Telemedicine Use by Age in Louisiana Medicaid During COVID-19: Claims-Based Longitudinal Analysis

**DOI:** 10.2196/46123

**Published:** 2023-04-26

**Authors:** Sooyeol Park, Brigham Walker, Andrew Anderson, Yixue Shao, Kevin Callison

**Affiliations:** 1 Department of Health Policy and Management Tulane University New Orleans, LA United States; 2 Murphy Institute for Political Economy Tulane University New Orleans, LA United States

**Keywords:** telemedicine, age, disparities, COVID-19, availability, health care services, infection, age, pandemic, usage, users, reliance, digital literacy, internet, trends

## Abstract

**Background:**

Limited availability of in-person health care services and fear of contracting COVID-19 during the pandemic promoted an increased reliance on telemedicine. However, long-standing inequities in telemedicine due to unequal levels of digital literacy and internet connectivity among different age groups raise concerns about whether the uptake of telemedicine has exacerbated or alleviated those inequities.

**Objective:**

The aim of this study is to examine changes in telemedicine and in-person health service use during the COVID-19 pandemic across age groups for Medicaid beneficiaries in the state of Louisiana.

**Methods:**

Interrupted time series models were used on Louisiana Medicaid claims data to estimate trends in total, in-person, and telemedicine monthly office visit claims per 1000 Medicaid beneficiaries between January 2018 and December 2020. Changes in care pattern trends and levels were estimated around the infection peaks (April 2020 and July 2020) and for an end-of-year infection leveling off period (December 2020). Four mutually exclusive age categories (0 to 17, 18 to 34, 35 to 49, and 50 to 64 years) were used to compare the differences.

**Results:**

Prior to the COVID-19 pandemic, telemedicine services accounted for less than 1% of total office visit claim volume across the age groups. Each age group followed similar patterns of sharp increases in April 2020, downward trends until sharp increases again in July 2020, followed by flat trends thereafter until December 2020. These sharp increases were most pronounced for older patients, with those aged 50 to 64 years seeing increases of 184.09 telemedicine claims per 1000 Medicaid beneficiaries in April 2020 (95% CI 172.19 to 195.99) and 120.81 in July 2020 (95% CI 101.32 to 140.31) compared with those aged 18 to 34 years, seeing increases of 84.47 (95% CI 78.64 to 90.31) and 57.00 (95% CI 48.21 to 65.79), respectively. This resulted in overall changes from baseline to December 2020 levels of 123.65 (95% CI 112.79 to 134.51) for those aged 50 to 64 years compared with 59.07 (95% CI 53.89 to 64.24) for those aged 18 to 34 years.

**Conclusions:**

Older Medicaid beneficiaries in Louisiana had higher rates of telemedicine claim volume during the COVID-19 pandemic compared with younger beneficiaries.

## Introduction

Limited availability of in-person health care services and concern over contracting COVID-19 during the initial phases of the pandemic prompted a rapid and widespread transition to telemedicine [1-4]. While telemedicine has the potential to reach patients of all ages and income levels, it remains unclear whether the rapid transition to telemedicine during the COVID-19 pandemic has altered care patterns differentially by age among underserved populations, including those with Medicaid coverage [5-10]. Several barriers related to telemedicine use, such as digital literacy, which tends to decline with age, have been identified and may inform differences in care patterns in the pandemic era [11,12].

Our objective in this study was to examine changes in telemedicine and in-person health service use during the COVID-19 pandemic across age groups for Medicaid beneficiaries in the state of Louisiana. Louisiana is well suited for our analysis as its residents generally experience more health challenges than people living in other states, and, as the only Medicaid expansion state in the Deep South, 40% of its population is covered through the Medicaid and Children’s Health Insurance Program. Though the state Medicaid program paid for telemedicine services at parity with in-person services prior to the COVID-19 pandemic, telemedicine use was uncommon. The first case of COVID-19 in Louisiana was confirmed on March 9, 2020, and within a matter of days, the state had the highest COVID-19 infection rate in the United States [13]. By the end of March, the Louisiana Department of Health had loosened restrictions on telemedicine service delivery, promulgated billing guidance for providers delivering telemedicine services to Medicaid beneficiaries, and directed providers to substitute telemedicine services for in-person care where appropriate [13-18].

A better understanding of telemedicine uptake by age for vulnerable populations will provide a basis for continued research on the prolonged effects of the pandemic and inform the active debate among policy makers centered on preserving pandemic-related changes to telemedicine regulations.

## Methods

### Data

This study used data from the Louisiana Medicaid Claims Data Warehouse from January 2018 to December 2020. Data were restricted to claims for evaluation and management (E&M) services, which have a high level of substitutability between in-person and telemedicine delivery. The sample was further restricted to those continuously enrolled in Louisiana Medicaid during the study period to eliminate any possible issues involving distributional changes in patient characteristics associated with the demand for care. Those dually eligible for Medicare and Medicaid coverage were excluded since the study team lacked access to Medicare claims. E&M services were identified using the following Current Procedural Terminology codes: 99201-99205 (“Office or other outpatient visits for the evaluation and management of a new patient,” ranging from 10 to 60 minutes of visit time), 99211-99215 (“Office or other outpatient visits for the evaluation and management of an established patient,” ranging from 5 to 40 minutes of visit time), and 99241-99245 (“Office consultation for a new or established patient,” ranging from straightforward to high complexity). In-person and telemedicine deliveries were identified using claim modifiers with GT, GQ, and 95 as telemedicine services or using a place of service code with 02, indicating a telemedicine modality for service delivery. We then evaluated trends in telemedicine use by creating four mutually exclusive age categories, which were 0-17, 18-34, 35-49, and 50-64 years. Our final sample was composed of claims for 850,821 Medicaid beneficiaries.

### Outcomes

Daily E&M claims were aggregated to the monthly level to minimize random variation and to provide a clear view of trends in service use. We then calculated monthly E&M service use per 1000 beneficiaries by dividing the total monthly E&M claims by the number of beneficiaries in our sample in each age category and multiplying by 1000. Following this procedure, we generated measures of all monthly E&M claims, monthly in-person E&M claims, and monthly telemedicine E&M claims per 1000 Medicaid beneficiaries for analysis.

Monthly pre–COVID-19 baseline E&M service use was calculated by generating an arithmetic mean of monthly pre–COVID-19 E&M claims per 1000 Medicaid beneficiaries from January 2018 to February 2020. Post–COVID-19 E&M service claims with and without telemedicine indicators were generated from April 2020 to December 2020.

### Statistical Analysis

Interrupted time series (ITS) models were used to estimate trends in total, in-person, and telemedicine monthly E&M claims per 1000 Medicaid beneficiaries. We included a linear monthly trend term to capture the pre–COVID-19 trend in E&M use and indicators for April 2020, July 2020, and December 2020. The first 2 periods represent the peaks of Louisiana’s first and second waves of COVID-19 infections, while the December 2020 indicator was included to capture use rates at the end of the pandemic’s first calendar year. We also included interaction terms between the monthly time trend and the indicators for April 2020 and July 2020 to estimate trend changes in the outcomes. Cumby-Huizinga tests were applied for autocorrelation, which led to the inclusion of a 2-period lag term in the models, and all models were estimated using ordinary least squares. We followed the Strengthening the Reporting of Observational Studies in Epidemiology (STROBE) reporting guidelines and were exempted from the Tulane Institutional Review Board.

### Ethical Considerations

This study was granted exempt status by Tulane Institutional Review Board (#2020-1945) and the Louisiana Department of Health Institutional Review Board.

## Results

**Figure 1:**
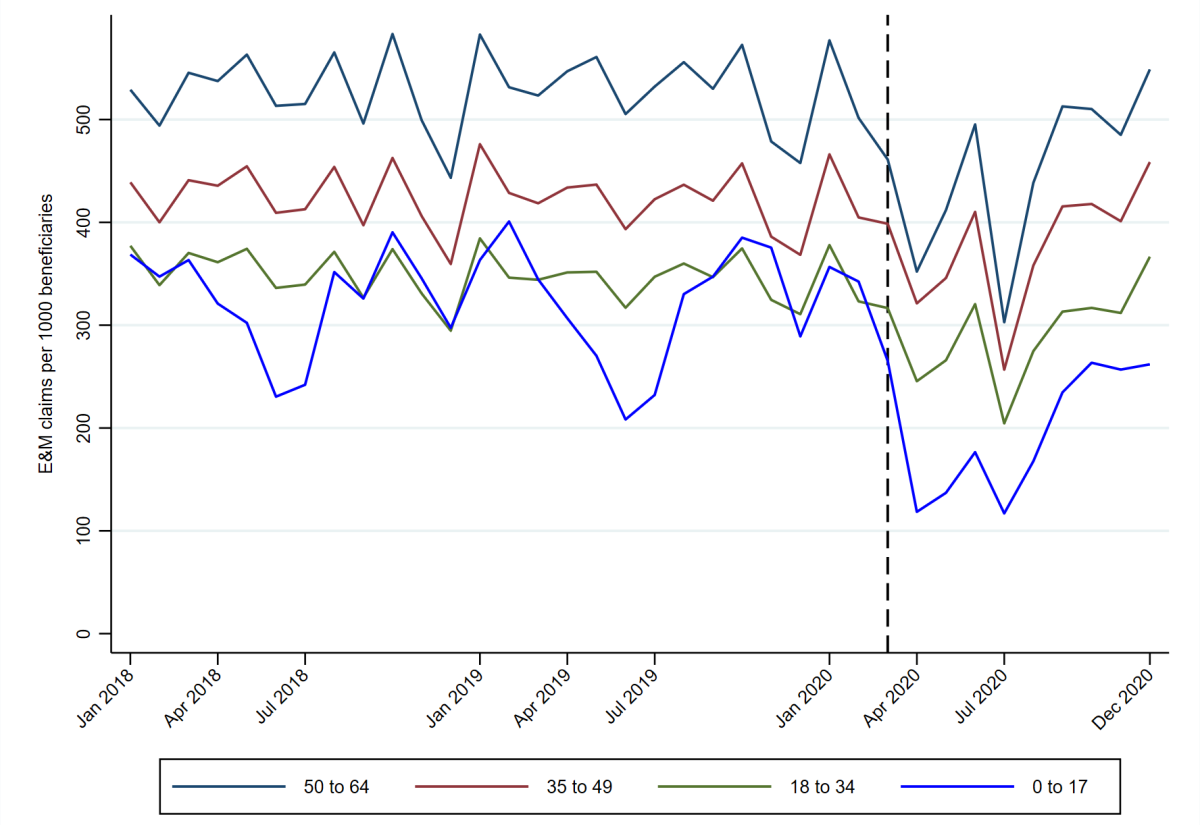
Trends in total evaluation and management (E&M) claims by age group.

Trends in total E&M claims by age group are shown in Figure 1, which plots monthly E&M claims per 1000 beneficiaries enrolled in Louisiana Medicaid by age. The vertical line represents March 2020, the month when stay-at-home orders were introduced in Louisiana and the Louisiana Department of Health expanded access to telemedicine services for Medicaid beneficiaries. Older beneficiaries used E&M services at a higher rate than those at younger ages both before and during the pandemic. All groups faced steep declines in E&M service use in April 2020, coinciding with the first wave of peak COVID-19 infections in Louisiana. Claim volume rebounded in May and June 2020 and then declined again with the second wave of peak COVID-19 infections in July 2020. After July 2020, E&M service use increased through the end of the year such that E&M claim volume for all age groups, except the 0-17–years group, had fully recovered by the end of 2020.

**Figure 2:**
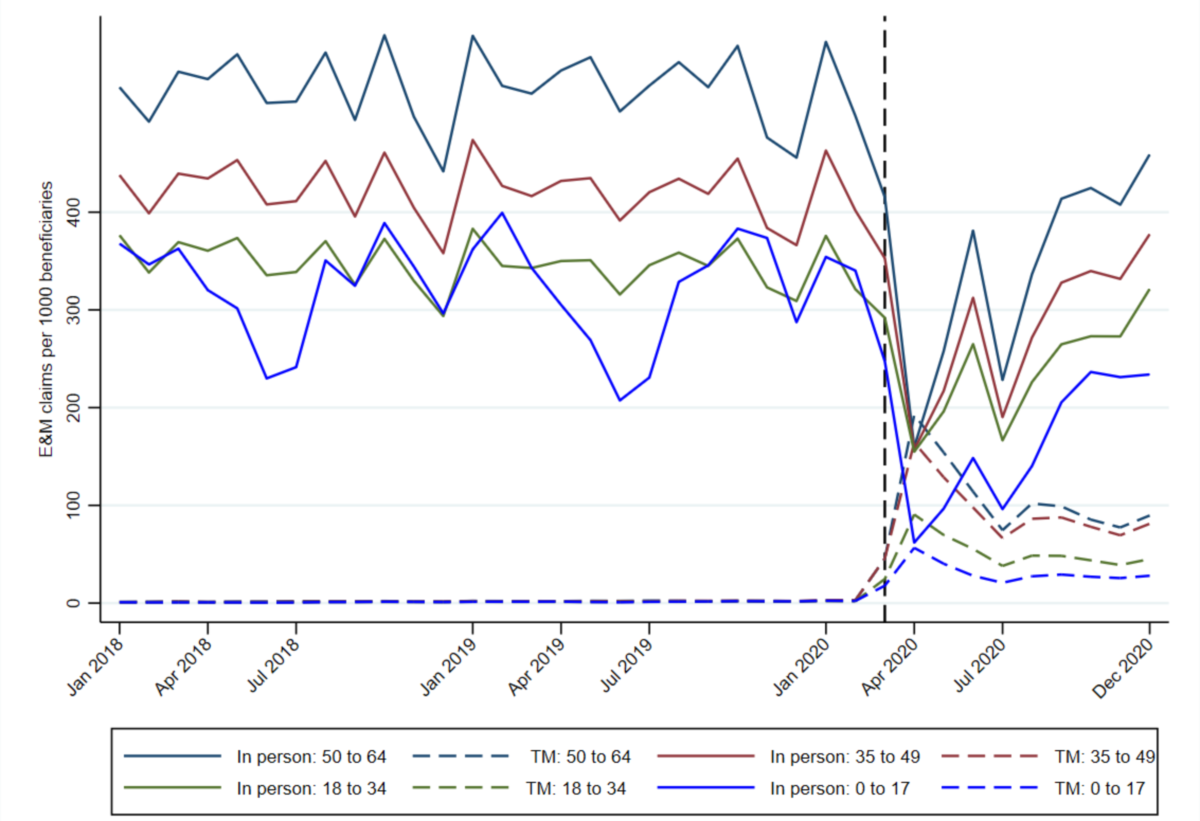
Trends in evaluation and management (E&M) claims by service type for age group. TM: telemedicine.

Figure 2 plots the trends in E&M claims by service modality (in-person and telemedicine) for each age group. The vertical line represents March 2020, the month when stay-at-home orders were introduced in Louisiana and the Louisiana Department of Health expanded access to telemedicine services for Medicaid beneficiaries. In-person E&M claims followed the same general pattern as overall claim volume with steep declines in April and July 2020 for all ages, while telemedicine claim volume spiked in April 2020. In-person claim volume, which had been consistently higher for those aged 50 to 64 years prior to the pandemic, fell to levels similar to those aged 35 to 49 years and those aged 18 to 34 years in April 2020. Telemedicine claim volume was highest for those aged 50 to 64 years in April 2020 and declined monotonically by age grouping. Notably, there was no spike in telemedicine use coinciding with the second wave of peak COVID-19 infections and a drop in in-person claim volume in July 2020. Telemedicine claim volume remained elevated compared to the prepandemic period for all age groups through December 2020.

Table 1 shows estimates from the ITS model corresponding to the graphical results in the figures. Baseline E&M claim volume increased with age such that those aged 50 to 64 years accrued approximately 200 claims per 1000 beneficiaries per month more than those aged 0 to 17 years (Table 1, column 1). Claim volume fell by 203.57 (95% CI –249.16 to –157.99) claims per 1000 beneficiaries aged 50 to 64 years with the first wave of peak COVID-19, a relative decrease of 36% compared to this group’s baseline average (Table 1, column 2). Beneficiaries aged 0 to 17 years accrued 184.61 (95% CI –219.39 to –149.84) fewer claims per 1000 compared to their baseline average, a relative decrease of 51%. Column 3 shows that E&M service use trended upward for all age groups in May and June 2020, after the first wave of peak COVID-19 infections had begun to subside.

Following this recovery, total E&M service use dropped by an even larger margin in July 2020 with the second wave of peak COVID-19 infections (Table 1, column 4). E&M claims began trending up in the months following this second wave, and by December 2020, most age groups had rebounded to baseline levels. Only those aged 0 to 17 years continued to experience dramatically reduced E&M claim volume by December 2020.

ITS estimates for changes in in-person E&M claim volume are presented in Table 2. Similar trends are observed with a substantial reduction in in-person claim volume coinciding with the first and second wave of peak COVID-19 infections in Louisiana (Table 2, columns 1 and 3). In-person E&M claim volume remained below baseline levels for all age groups as of December 2020.

Table 3 includes ITS estimates for changes in telemedicine E&M claim volume. Prior to the pandemic, telemedicine services accounted for less than 1% of total E&M claim volume for all age groups. Telemedicine E&M claim volume spiked in April 2020, coinciding with the first wave of peak COVID-19 infections in Louisiana and the Louisiana Department of Health’s guidance to providers to substitute telemedicine services for in-person services when appropriate to do so. Beneficiaries aged 50 to 64 years saw both the largest absolute increase in telemedicine claim volume (184.09 claims per 1000 members; 95% CI 172.19 to 195.99) and the largest relative increase, a 70-fold gain over baseline levels. Relative increase in telemedicine use for the other age groups ranged from a 60-fold increase for those aged 35 to 49 years to a 30-fold increase for those aged 0 to 17 years. Telemedicine claim volume began to wane as the first peak wave of infections subsided, and, though telemedicine claim volume remained well above baseline averages, there was no observed spike in claims coinciding with the second wave of peak COVID-19 infections in July 2020. By December 2020, telemedicine claim volume was below its April 2020 peak, despite rising infections from the Beta variant, but remained above baseline levels for all age groups.

**Table 1:** Estimates of changes in total evaluation and management service use by age group.

Age group	Time span^a^					
	(1)^b^	(2)^c^	(3)^d^	(4)^e^	(5)^f^	(6)^g^
	Baseline trend (January 2018 to February 2020)	April 2020; change from baseline	April 2020 to June 2020 trend	July 2020; change from baseline	July 2020 to November 2020 trend	December 2020; changes from baseline
50 to 64 years	562.68 (520.53, 604.84)	–203.57 (–249.16, –157.99)	87.82 (66.16, 109.48)	–266.28 (–341,25, –191.31)	48.65 (26.84, 70.46)	–36.83 (–87.36, 13.69)
35 to 49 years	460.02 (431.11, 488.93)	–125.29 (–156.81, –93.77)	60.95 (40.18, 81.72)	–188.71 (–245.54, –131.88)	37.4 (21.17, 53.72)	–1.44 (–40.26, 37.38)
18 to 34 years	378.87 (366.41, 391.33)	–112.08 (–139.65, –84.51)	51.65 (34.58, 68.71)	–163.13 (–204.67, –121.58)	27.27 (17.17, 37.36)	–8.55 (–46.12, 29.01)
0 to 17 years	361.17 (350.61, 371.72)	–184.61 (–219.39, –149.84)	75.11 (61.52, 88.70)	–198.73 (–236.58, –160.88)	7.02 (–4.94, 19.00)	–101.88 (–128.90, –74.82)

^a^95% CIs intervals are in parentheses. Regression estimates are from an interrupted time series specification that includes a monthly time trend; indicators for April 2020, July 2020, and December 2020; and interactions between the trend term and the April and July 2020 indicators. Data for each regression model comprise 36 month-year level observations. Cumby-Huizinga tests for autocorrelation led to the inclusion of a maximum lag of order 2.

^b^Baseline average monthly use rates per 1000 beneficiaries from January 2018 to February 2020.

^c^Coefficient estimate for the April 2020 indicator.

^d^Sum of the monthly time trend and the coefficient estimate of the interaction between the trend term and the April 2020 indicator.

^e^Difference between the estimate for July 2020 use and the baseline average.

^f^Sum of the monthly time trend and the coefficient estimate of the interaction between the trend term and the July 2020 indicator.

^g^Difference between the estimate for December 2020 use and the baseline average.

**Table 2:** Estimates of changes in in-person evaluation and management service use by age group.

Age group	Time span^a^					
	(1)^b^	(2)^c^	(3)^d^	(4)^e^	(5)^f^	(6)^g^
	Baseline trend (January 2018 to February 2020)	April 2020; change from baseline	April 2020 to June 2020 trend	July 2020; change from baseline	July 2020 to November 2020 trend	December 2020; changes from baseline
50 to 64 years	560.05 (513.37, 606.73)	–387.68 (–443.17, –332.18)	126.81 (104.27, 149.35)	–387.10 (–456.11, –318.09)	49.32 (33.18, 65.47)	–160.48 (–214.24, –106.73)
35 to 49 years	457.33 (424.16, 490.50)	–279.95 (–318.26, –241.65)	93.84 (73.92, 113.75)	–291.72 (–342.79, –240.64)	37.33 (25.67, 48.99)	–109.49 (–150.29, –68.69)
18 to 34 years	377.09 (361.94, 392.23)	–196.57 (–226.91, –166.22)	69.01 (53.32, 84.70)	–220.14 (–259.50, –180.77)	27.43 (19.20, 35.66)	–67.62 (–106.48, –28.76)
0 to 17 years	359.35 (346.87, 371.82)	–236.13 (–273.43, –198.83)	88.99 (76.21, 101.76)	–233.02 (–274,43, –191.61)	6.09 (–6.87, 19.04)	–139.65 (–168.45, –110.84)

^a^95% CIs are in parentheses. Regression estimates are from an interrupted time series specification that includes a monthly time trend; indicators for April 2020, July 2020, and December 2020; and interactions between the trend term and the April and July 2020 indicators. Data for each regression model comprise 36 month-year level observations. Cumby-Huizinga tests for autocorrelation led to the inclusion of a maximum lag of order 2.

^b^Baseline average monthly use rates per 1000 beneficiaries from January 2018 to February 2020.

^c^Coefficient estimate for the April 2020 indicator.

^d^Sum of the monthly time trend and the coefficient estimate of the interaction between the trend term and the April 2020 indicator.

^e^Difference between the estimate for July 2020 use and the baseline average.

^f^Sum of the monthly time trend and the coefficient estimate of the interaction between the trend term and the July 2020 indicator.

^g^Difference between the estimate for December 2020 use and the baseline average.

**Table 3:** Estimates of changes in telemedicine evaluation and management service use by age group.

Age group	Time span^a^					
	(1)^b^	(2)^c^	(3)^d^	(4)^e^	(5)^f^	(6)^g^
	Baseline trend (January 2018 to February 2020)	April 2020; change from baseline	April 2020 to June 2020 trend	July 2020; change from baseline	July 2020 to November 2020 trend	December 2020; changes from baseline
50 to 64 years	2.63 (–2.82, 8.09)	184.09 (172.19, 195.99)	–38.98 (–41.62, –36.33)	120.81 (101.32, 140,31)	–0.66 (–7.14, 5.80)	123.65 (112.79, 134.51)
35 to 49 years	2.69 (–2.69, 8.08)	154.66 (143.26, 166.05)	–32.88 (–35.64, –30.12)	103.00 (86.46, 119.53)	0.12 (–5.14, 5.38)	108.05 (98.12, 117.97)
18 to 34 years	1.78 (–1.13, 4.70)	84.47 (78.64, 90.31)	–17.35 (–19.19, –15.51)	57.00 (48.21, 65.79)	–0.15 (–2.87, 2.55)	59.07 (53.89, 64.24)
0 to 17 years	1.81 (–0.11, 3.75)	51.51 (47.46, 55.55)	–13.87 (–15.11, –12.63)	34.28 (28.89, 39.67)	0.93 (–0.64, 2.52)	37.76 (34.35, 41.16)

^a^95% CIs intervals are in parentheses. Regression estimates are from an interrupted time series specification that includes a monthly time trend; indicators for April 2020, July 2020, and December 2020; and interactions between the trend term and the April and July 2020 indicators. Data for each regression model comprise 36 month-year level observations. Cumby-Huizinga tests for autocorrelation led to the inclusion of a maximum lag of order 2.

^b^Baseline average monthly use rates per 1000 beneficiaries from January 2018 through February 2020.

^c^Coefficient estimate for the April 2020 indicator.

^d^Sum of the monthly time trend and the coefficient estimate of the interaction between the trend term and the April 2020 indicator.

^e^Difference between the estimate for July 2020 use and the baseline average.

^f^Sum of the monthly time trend and the coefficient estimate of the interaction between the trend term and the July 2020 indicator.

^g^Difference between the estimate for December 2020 use and the baseline average.

## Discussion

### Principal Findings

Telemedicine served a critical role in minimizing service disruptions during the first year of the COVID-19 pandemic, and physicians and patients both reported positive experiences with its use [11,19]. There is some evidence to suggest that the transition to telemedicine during the pandemic allowed physicians to preserve care continuity, ease the provider shortage in rural areas, and reduce health care costs [20,21]. However, there remain concerns over the rapid transition to telemedicine during the COVID-19 pandemic owing to long-standing inequities in telemedicine access, including unequal levels of digital literacy and broadband internet connectivity along the age distribution [11,22-28].

Total and in-person E&M claim volume declined steeply in April and July 2020, coinciding with the first 2 peak waves of COVID-19 infections in Louisiana. Telemedicine use increased across all age groups beginning in April 2020. This finding is consistent with previous studies, which reported reduced in-person visits and increased use of telemedicine during the COVID-19 pandemic in Medicare populations [2,29,30]. Older Medicaid beneficiaries also experienced a relatively larger increase in E&M telemedicine claim volume compared with younger Medicaid beneficiaries following the onset of the pandemic. The oldest age group in our study, those aged 50-64 years, saw the largest increase in telemedicine use during the first wave of peak COVID-19 infections. The trend of higher telemedicine use among older age groups continued through the end of 2020, with those aged 50 to 64 years exhibiting the highest sustained rate of telemedicine claim volume. The youngest age group in our sample, those aged 0 to 17 years, saw the smallest increase in telemedicine claim volume and was the only age group that had not recovered to their pre–COVID-19 E&M claim level by December 2020.

Prior studies have reported mixed findings on the association between age and telemedicine use [5,26,31-37]. However, these studies differ from ours in several important ways. First, prior studies have generally examined the extensive margin of telemedicine use (ie, ever having used telemedicine) as opposed to the quantity or extensiveness of its use, which is captured in our claim volume measure [5,35,37,38]. Second, studies have typically focused on patients at a single medical center or those seeking care from narrow subsets of providers (eg, otolaryngologists) [35,37,38]. By contrast, our study examines a broad patient population receiving all manner of evaluation and management services. Third, we included data through December 2020, capturing both the initial wave of COVID-19 infections that peaked in April 2020 and the second wave that peaked in July 2020. Prior studies that have relied on pandemic-era data limited to the initial months following onset lack insight into the characteristics of telemedicine patients during peak telemedicine use periods [5]. Finally, the study closest to ours in terms of scope and research setting examined rates of telemedicine use among commercially insured patients through July 2020 for office visits, including E&M services, and reported higher rates of telemedicine use among older adults (aged >46 years) compared with younger adults (aged 19-45 years), adolescents (aged 13-18 years), and children (aged 0-12 years) [6].

To our knowledge, this is the first study to assess trends and differences in telemedicine use by age for a panel of Medicaid beneficiaries during the COVID-19 pandemic. State Medicaid programs dramatically expanded the scope of covered services available to be delivered through telemedicine in the early stages of the COVID-19 pandemic, and many of those policies remain in place today [39]. However, researchers and patient advocates have voiced concern over the equity implications of expanded reliance on telemedicine and have called attention to the fact that some populations, including older adults, face critical barriers in accessing telemedicine services [40,41]. Our findings can inform this policy debate by highlighting the link between the transition to telemedicine services during the pandemic and access to care for older Medicaid beneficiaries. The fact that older Medicaid beneficiaries in Louisiana saw a higher share of E&M claim volume delivered through telemedicine compared with younger beneficiaries indicates that the expanded availability of telemedicine was an important tool for maintaining access to care during the early peak waves of COVID-19 infections. Furthermore, the continued higher rates of telemedicine use for older adults in December 2020, after stay-at-home orders and other mitigation policies had expired in Louisiana, signifies the potential for telemedicine to serve as an avenue to maintain increased flexibility and access to care.

### Limitations

Despite the novelty of our findings and their implications for Medicaid policy, our research has several important limitations. Identification in our ITS models was driven by trend breaks in the outcome measures, and we cannot rule out the effect of confounders unrelated to the pandemic [38]. However, it is unlikely that the changes we observed were driven by factors other than COVID-19, as the steep decline in E&M service use coincided with the introduction of physical distancing orders and telemedicine guidance in Louisiana. Second, this study was based on Louisiana Medicaid beneficiaries, and the findings may not generalize to other population settings with divergent patterns of medical use. Finally, we were unable to distinguish between video and telephonic telemedicine visits. Insofar as digital literacy may differentially impact older beneficiaries’ access to video but not telephonic services, we cannot provide evidence related to this distinction.

### Conclusion

Older Medicaid beneficiaries in Louisiana had higher rates of telemedicine claim volume for E&M services during the COVID-19 pandemic compared with younger beneficiaries. These findings can help guide policy makers as they continue to debate the merits of extending COVID-19–era flexibilities related to telemedicine use and service provision. Future research should assess whether the increased use of telemedicine has resulted in better long-term access and care quality for Medicaid beneficiaries.

## Abbreviations

E&M: evaluation and management
ITS: interrupted time series
STROBE: Strengthening the Reporting of Observational Studies in Epidemiology
